# Outcome for Advanced or Metastatic Soft Tissue Sarcoma of Nonextremities Treated with Doxorubicin-Based Chemotherapy: A Retrospective Study from a Single Cancer Institution

**DOI:** 10.1155/2018/8926598

**Published:** 2018-04-18

**Authors:** Shoko Marshall, Kenji Nakano, Yoshiya Sugiura, Shinichiro Taira, Makiko Ono, Junichi Tomomatsu, Shunji Takahashi

**Affiliations:** ^1^Department of Medical Oncology, Cancer Institute Hospital, Japanese Foundation for Cancer Research, Tokyo, Japan; ^2^Department of Pathology, Cancer Institute Hospital, Japanese Foundation for Cancer Research, Tokyo, Japan

## Abstract

**Background:**

Doxorubicin is the key drug for treatment of advanced soft tissue sarcoma (STS). The appropriate dosage of doxorubicin, regarding monotherapy or the role of combination therapy, is unclear.

**Methods:**

We retrospectively reviewed patients with advanced or metastatic STS of nonextremities who were treated with doxorubicin-based chemotherapies in our institution. Time to treatment failure (TTF), overall survival (OS), overall response, and prognostic factors for OS were evaluated.

**Results:**

Seventy-five patients were enrolled. The median TTF was 4.7 months, and the median OS was 20.1 months. The overall response rate was 20%. Doses of doxorubicin monotherapy did not show significant difference either in TTF or in OS. There were no significant differences in OS between combination therapy and monotherapy, but the TTF with combination therapy was better than monotherapy. The overall response for combination therapy indicated a better response rate. Less number of involved organs, no bulky mass, and a normal CRP level were independent favorable prognostic factors for OS.

**Conclusions:**

Combination therapy showed better response and TTF than monotherapy but did not show better OS. Possible prognostic factors for OS were indicated. This retrospective study was approved by the institutional review board. This trial is registered with UMIN000028787.

## 1. Introduction

Soft tissue sarcomas (STSs) are a rare form of cancer which originate in various sites of the body [1]. STSs originating in the nonextremities can involve various sites of the body, and complete surgical resection can be difficult in some cases. Therefore, STSs of nonextremities origin are reported to have a worse prognosis when compared to STSs originating in the extremities [2, 3]. Systemic chemotherapy is often administered, and doxorubicin is currently a key drug used for treatment of advanced STS. Doxorubicin monotherapy and combination chemotherapy containing doxorubicin are options for treating advanced or metastatic STSs. The optimal dose of doxorubicin in regard to doxorubicin monotherapy remains unclear, and the role of combination therapy is controversial. The purpose of this study is to reveal the optimal dose of doxorubicin and the role that combination therapy plays in STSs originating in the nonextremities. The other purpose of this study is to reveal the clinical factors which predict prognosis in doxorubicin therapy for advanced or metastatic STS of nonextremities.

## 2. Materials and Methods

We retrospectively reviewed patients with advanced or metastatic STS of nonextremities who received doxorubicin-based chemotherapies at the Department of Medical Oncology in our institution from October 2005 to April 2016. A histopathological diagnosis was done by a biopsy or surgery which was reviewed by a well-trained pathologist in our institution. The regimens included a single agent of doxorubicin, a combination mostly with cyclophosphamide/vincristine/dacarbazine (CYVADIC), or ifosfamide (AI). The dose of doxorubicin monotherapy varied from 60 mg/m^2^ to 75 mg/m^2^, and CYVADIC consisted of 50 mg/m^2^ doxorubicin, 1.5 mg/m^2^ vincristine (max 2.0 mg/body, Day 1), 250 mg/m^2^ dacarbazine (Day 1–5), and 500 mg/m^2^ cyclophosphamide (Day 2), and AI consisted of 30 mg/m^2^ doxorubicin (Day 1–2) and 2 g/m^2^ ifosfamide (Day 1–5). The treatment regimen and the doxorubicin dose were chosen by a physician's choice. The doxorubicin monotherapy was chosen for the purpose of extending the patient's life, whereas the combination therapy was chosen with the purpose of shrinking the tumor. The patient's age, PS, organ functions, and risks such as the risk of myelosuppression were taken into consideration when determining the treatment regimen and the dose of chemotherapy. Clinical evidence in regard to the benefit of OS is based on some randomized clinical trials, such as EORTC62012. These trials in turn possibly affected the preference of physicians' choices regarding the use of doxorubicin monotherapy. The doxorubicin-based chemotherapy was discontinued until progressive disease (PD), unacceptable adverse events, or the cumulative dose was reached up to 450 mg/m^2^. Imaging studies were performed every 2 to 3 months, or whenever the patients' presented with exacerbated symptoms. Objective responses, according to Response Evaluation Criteria in Solid Tumors (RECIST) version 1.1, were assessed by computed tomography (CT) or magnetic resonance imaging (MRI). Time to treatment failure (TTF) and overall survival (OS) were assessed by the Kaplan–Meier method. A univariate log-rank analysis was used to assess potential prognostic factors for OS, and the independent significant factors were investigated by multivariate Cox regression analyses for which the *p* value was less than 0.05.

## 3. Results

Between October 2005 and April 2016, a total of 75 patients were enrolled for analysis. The baseline characteristics of the patients treated with the doxorubicin-based chemotherapy are shown in Table 1. The patients consisted of 53% males, and the median age was 55 years (range: 21–75). Most of the patients had good performance status, and only 3 patients had ECOG performance status of 2. The locations of the primary tumor were the head and neck (19%), the thorax (9%), the abdomen (31%), the retroperitoneum (25%), the genital organs (5%), and others (5%). Others included breast, subcutaneous, groin, and the back. Regarding the number of involved organs, 29% of patients had only 1 involved organ and 36% of patients had more than 3 involved organs. The sites of the involved organs when doxorubicin therapy was administered included the head and neck (15%), intra-abdomen (40%), retroperitoneum (20%), lungs (47%), liver (29%), bone (24%), and lymph nodes (21%). Other sites of involved organs include the adrenal glands, subcutaneous, muscles, the pancreas, the renal, the spleen, the heart, the intestine, the ovaries, the uterus, the prostate, and the bladder. The median maximum length of the tumor when doxorubicin-based chemotherapy was initiated was 5.3 cm (range: 0–21.7), and 53% had a mass which was measured to be more than 5 cm in diameter. The majority of patients received pretreatment consisting of mostly surgery (65%), radiation therapy (28%), and systemic therapy such as imatinib, sunitinib, gemcitabine, a combination of gemcitabine and docetaxel, ifosfamide, paclitaxel, and AI (11%). However, 24% of the patients had no treatment for advanced diseases before the doxorubicin-based chemotherapy. Histopathologically, 23% of the patients were diagnosed with leiomyosarcoma and 20% were diagnosed with liposarcoma, respectively, followed by spindle cell sarcoma (19%), pleomorphic sarcoma (8%), and synovial sarcoma (7%). The others were diagnosed as angiosarcoma, undifferentiated pleomorphic sarcoma, solitary fibrous tumor, low-grade fibromyxoid sarcoma, malignant peripheral nerve sheath tumor, breast phyllodes tumor, round cell sarcoma with CIC rearrangement, neuroblastoma, malignant extrarenal rhabdoid tumor, and myxofibrosarcoma. Doxorubicin monotherapy was performed on 51% of the patients, including 55% of the patients with 60 mg/m^2^ and 42% with 75 mg/m^2^. One patient received 50 mg/m^2^ doxorubicin as a single agent due to having a previous medical history of a brain hemorrhage. Forty-nine percent of the patients had combination therapy, mostly with CYVADIC (81%), and AI was administered to 14%. The baseline characteristics of patients according to combination therapy or dose of doxorubicin were similar, except for the median age; the patients who had combination therapy were younger (median age 50 years, range: 21–74) compared to those with doxorubicin monotherapy (63 years, range: 24–72). The median total dose of doxorubicin was 300 mg/m^2^.

**Figure 1:**
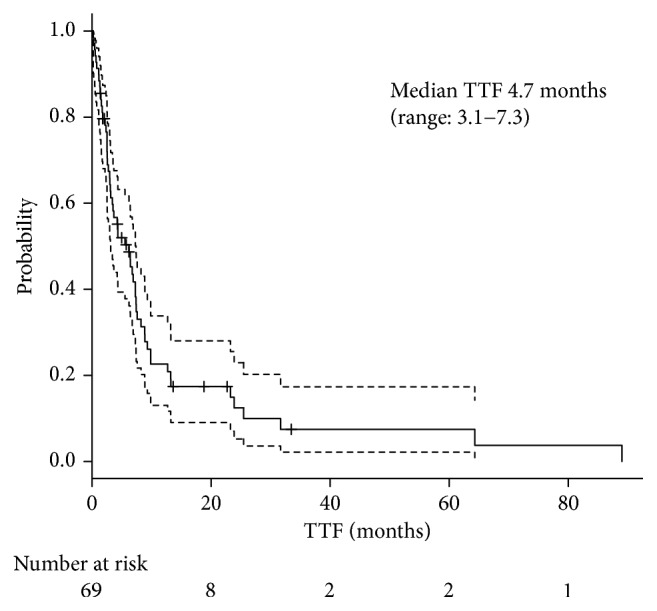
Median TTF.

**Figure 2:**
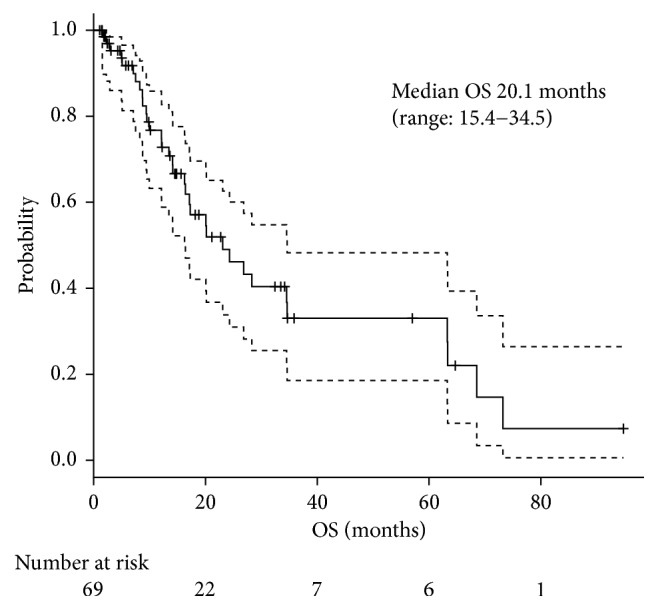
Median OS.

The median follow-up time was 14.3 months. Of the enrolled patients, 48 were alive and 15% were maintaining the treatment effect or under the treatment at the data cutoff of April 2016. Nine percent were keeping more than stable disease (SD) after the doxorubicin-based chemotherapy, including one partial response (PR) and one complete response (CR), respectively. One patient showed PR after the treatment but moved on to the second-line therapy, without break by the physician's decision. The median TTF was 4.7 months (Figure 1), and the median OS was 20.1 months (Figure 2). The overall response rate was 20%, including CR with 4%, and they all were treated with CYVADIC therapy. Forty-one percent had SD, and 36% had PD (Table 2). Out of 64 patients who discontinued the chemotherapy with doxorubicin, 66% received post-anticancer therapy. An operation was performed on 7%, 29% had radiation therapy, and 76% received systemic therapy. One patient with retroperitoneum liposarcoma underwent a resection of peritoneal dissemination twice, 4 and 6 months after doxorubicin chemotherapy. Another patient with a malignant rhabdoid tumor with liver and bilateral ovaries metastasis underwent resection of pubic tumor twice, 4 and 9 months after administering 5 courses of CYVADIC. The other patient with synovial sarcoma with multiple lung metastases had 4 courses of CYVADIC and underwent a partial resection of both the right and left lung and resection of the left upper lobe. The patient also had a resection of liver metastasis 6 months later. These patients had a relapse a few months after their last surgery. Postsystemic therapy included pazopanib (40%), ifosfamide (17%), irinotecan (17%), gemcitabine (7%), and other agents. Reasons for doxorubicin-based chemotherapy discontinuation were mostly due to progressive disease (94%); only 2 patients (3%) stopped the treatment because of adverse events (decreased cardiac function and anorexia), and 2 patients (3%) stopped the treatment due to the patient's request.

**Figure 3:**
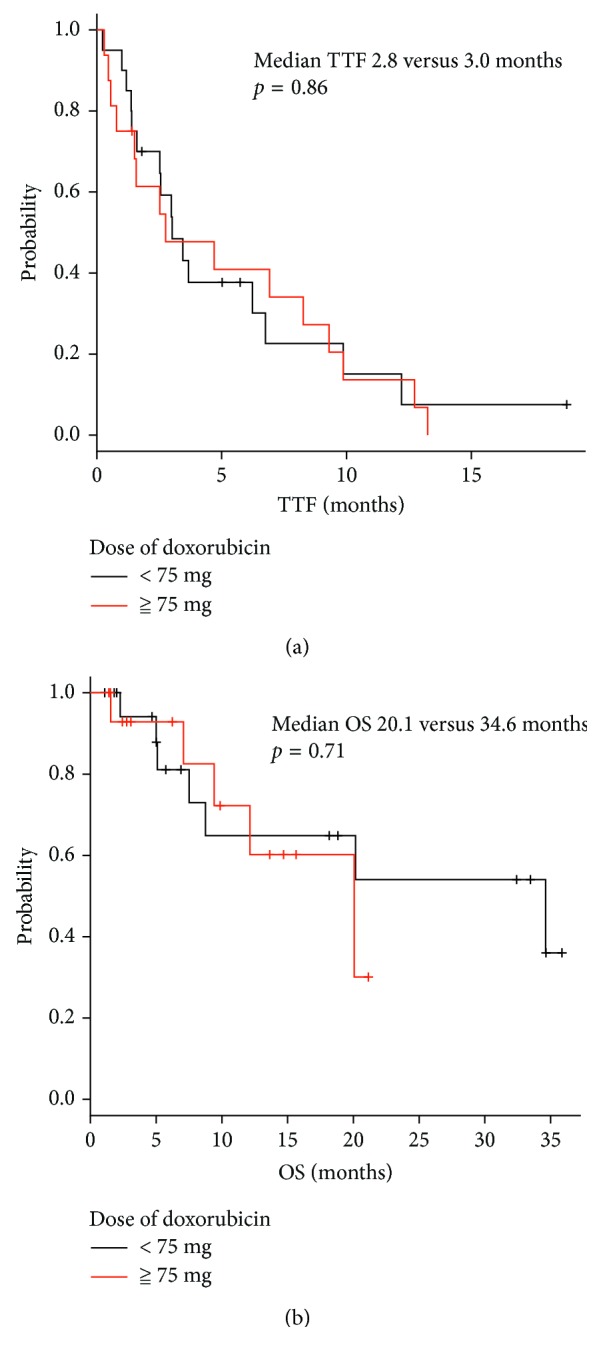
Difference of (a) TTF and (b) OS in doses of doxorubicin monotherapy.

**Figure 4:**
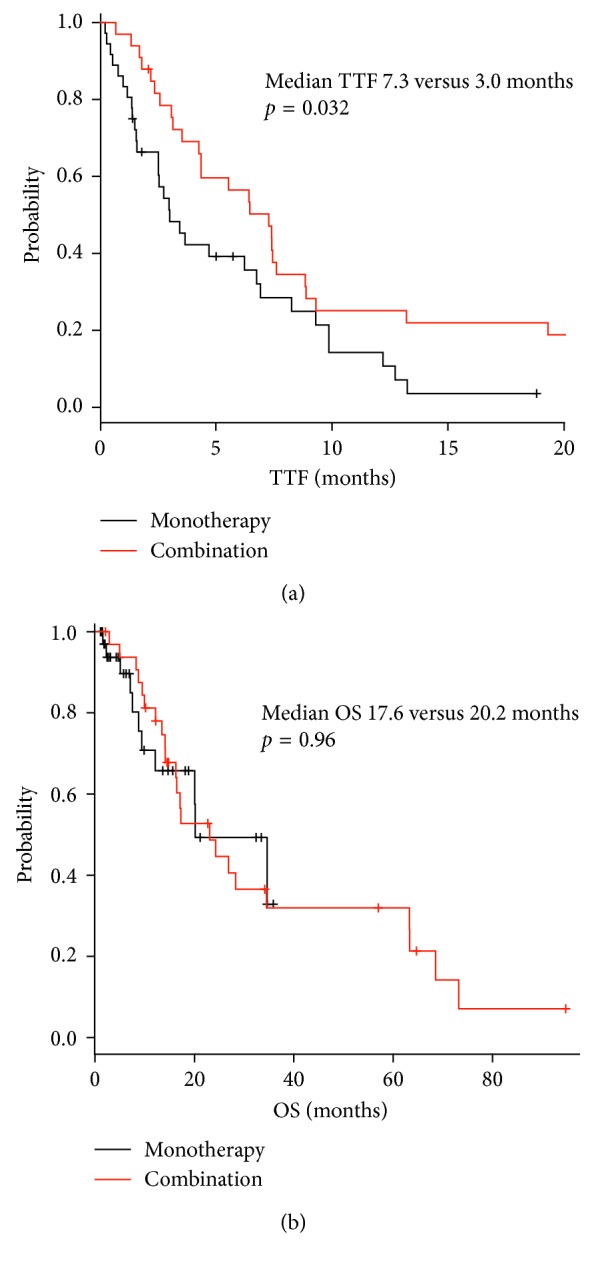
Difference of (a) TTF and (b) OS in combination therapy and monotherapy.

Doses of doxorubicin monotherapy administering 75 mg/m^2^ or less than 75 mg/m^2^ did not show significant difference either in TTF or in OS (Figure 3). The tumor response for different doses of doxorubicin monotherapy was also similar between 75 mg/m^2^ and less than 75 mg/m^2^ (Table 3). There were no significant differences in OS between doxorubicin-based combination therapy and doxorubicin monotherapy, but TTF of patients treated with combination therapy was better than doxorubicin monotherapy (Figure 4). The overall response for combination therapy was 30% compared to 11% doxorubicin monotherapy showing a better response rate (Table 4). Sex, age, PS, number of involved organs (≧3 organs), sites of involved organs, bulky mass (≧5 cm), presence of previous treatment, neutrophil to lymphocyte ratio (NLR), Hb (<11.6 g/dl), LDH (≧222 mg/dl), ALP (≧322 IU/l), and CRP (≧0.14 mg/dl) were investigated as prognostic factors for OS by univariate analysis (Table 5). Good PS (PS 0), less number of involved organs (<3), no bulky mass, and normal CRP level were favorable prognostic factors for OS. On multivariate analysis, less number of involved organs, no bulky mass, and normal CRP level were independent favorable prognostic factors, with a hazard ratio of 0.31 (95% CI: 0.15–0.65, *p*=0.0019), 0.27 (95% CI: 0.12–0.60, *p*=0.0013), and 0.43 (95% CI: 0.20–0.93, *p*=0.032), respectively (Table 5).

## 4. Discussion

Locally advanced or metastatic STS of nonextremities is generally considered to be incurable and has poor prognosis. Salvage therapies such as systemic therapy and operation and radiation therapies are usually performed to such patients, and doxorubicin remains the most active single agent in STS for systemic chemotherapy. However, the optimal dose of doxorubicin in regard to doxorubicin monotherapy is unclear, neither is the role of combination therapy, especially when it is restricted to nonextremities STS. A number of trials comparing doxorubicin as a single agent with combination chemotherapy were performed, and a few studies showed a better overall response rate. However, according to those trials, combination chemotherapy has not indicated survival advantage compared to a single-agent chemotherapy [4–7]. In our analysis, median TTF was 4.7 months, and median OS was 20.1 months, with an overall response of 20%. Sixty-six percent of patients had posttreatment and among those patients, 36% had surgery or radiotherapy, which was comparatively high. Some of the cases were metastatic, but it is possible that oligometastatic cases in which salvage surgeries could be the option were included. This inclusion of oligometastatic cases could have contributed to our OS. Combination therapy showed a better overall response and longer TTF, but neither combination therapy nor difference in doses of doxorubicin monotherapy showed any significant difference in OS, which was in agreement with previous trials. Most of the patients discontinued doxorubicin-based chemotherapy because of PD, and there were only two cases which stopped the chemotherapy because of adverse effects. We also compared doses of a single agent of doxorubicin (75 mg/m^2^ or less than 60 mg/m^2^), but there was no significant difference in TTF nor in OS. We tried to reveal prognostic factors for OS. There have been previously reported favorable and unfavorable prognostic factors. For example, Van Glabbeke et al. reported good PS, absence of liver metastases, long time lapse since initial diagnosis, and young age as favorable prognostic factors of survival time for advanced patients treated with anthracycline-containing regimens [8]. There is also a report that found young age, liposarcoma, and synovial histology as favorable prognostic factors and bone involvement as an unfavorable prognostic factor for those with advanced soft tissue sarcoma treated on palliative therapy [9]. Lymphopenia is also reported as an unfavorable prognostic factor for sarcomas and other advanced carcinomas [10]. We included sex, age, PS, number of involved organs, sites of involved organs, bulky mass, pretreatment, and NLR, Hb, LDH, ALP, and CRP levels to investigate for prognostic factors, and on multivariate analysis, less number of involved organs, no bulky mass, and normal CRP level were found to be favorable prognostic factors in regard to OS. In our nonextremities advanced or metastatic cases, most of the patients had good PS; therefore, PS did not show as a prognostic factor, and sites of organs were not found to be positive, but the number of involved organs and bulky mass were relevant as prognostic factors. The three favorable prognostic factors, identified in this study, are considered of value in point of restricting to advanced or metastatic STS of nonextremities.

We acknowledge that there are several limitations in this study since this was a retrospective study. Some of the decisions were made by individual physicians. For example, when imaging studies were performed, they may influence TTF. Furthermore, chemotherapy regimens and the dose of chemotherapy were chosen by a physician's choice. As a result, the dose of doxorubicin, in our study, was lower than the standard dose, in a high proportion of the patients. A further investigation which includes a prospective randomized study is needed.

## 5. Conclusion

In conclusion, doses of doxorubicin and doxorubicin monotherapy or combination therapy did not show significant differences in OS, but combination therapy showed a better overall response rate and longer TTF compared to monotherapy. Furthermore, less number of involved organs, no bulky mass, and normal CRP level were found to be independent favorable prognostic factors.

## Figures and Tables

**Table 1:** The baseline characteristics of the patients treated with the doxorubicin-based chemotherapy.

	No. (%)
*Sex*	
Male	40 (53)
Female	35 (46)
*Age*	
Median age, years	55 (21–75)
*ECOG performance status*	
0	57 (76)
1	15 (20)
2	3 (4)
*Location of the primary tumor*	
Head and neck	14 (19)
Thorax	7 (9)
Abdomen	23 (31)
Retroperitoneum	19 (25)
Genital organs	4 (5)
Others	4 (5)
Unknown	4 (5)
*Number of involved organs*	
1	22 (29)
2	26 (35)
3	13 (17)
4	10 (13)
≧5	4 (5)
*Site of involved organs, no*. (*%*)	
Head and neck	11 (15)
Intra-abdomen	30 (40)
Retroperitoneum	15 (20)
Lung	35 (47)
Liver	22 (29)
Bone	18 (24)
Lymph node	16 (21)
Others	29 (39)
*Maximum length*	
Median (cm)	5.3 (0–21.7)
>5 cm	40 (53)
*Pretreatment*	
Operation	49 (65)
Radiation	21 (28)
Chemotherapy	8 (11)
None	18 (24)
*Histopathology type*	
Leiomyosarcoma	17 (23)
Liposarcoma	15 (20)
Spindle cell sarcoma, NOS	14 (19)
Pleomorphic sarcoma	6 (8)
Synovial sarcoma	5 (7)
Others^∗^	18 (24)

^∗^Others include angiosarcoma, undifferentiated pleomorphic sarcoma, solitary fibrous tumor, low-grade fibromyxoid sarcoma, malignant peripheral nerve sheath tumor, breast phyllodes tumor, round cell sarcoma with CIC rearrangement, neuroblastoma, malignant extrarenal rhabdoid tumor, and myxofibrosarcoma.

**Table 2:** Overall response rate.

Best overall response	No. (%)
Complete response	3 (4)
Partial response	12 (16)
Overall response	15 (20)
Stable disease	31 (41)
Progressive disease	27 (36)
Not evaluable or not assessed	2 (3)

**Table 3:** Tumor response for different doses of doxorubicin monotherapy.

Best overall response	Doxorubicin ≧ 75 mg/m^2^ (*n* = 16), no. (%)	Doxorubicin < 75 mg/m^2^ (*n* = 22), no. (%)
Complete response	0 (0)	0 (0)
Partial response	2 (13)	2 (6)
Overall response	2 (13)	2 (6)
Stable disease	5 (31)	14 (44)
Progressive disease	8 (50)	6 (19)
Not evaluable or not assessed	1 (6)	0 (0)

**Table 4:** Overall response rate for doxorubicin monotherapy and combination therapy.

Best overall response	A single agent (*n* = 38), no. (%)	Combination therapy (*n* = 37), no. (%)
Complete response	0 (0)	3 (8)
Partial response	4 (11)	8 (22)
Overall response	4 (11)	11 (30)
Stable disease	19 (50)	12 (32)
Progressive disease	14 (37)	13 (35)
Not evaluable or not assessed	1 (3)	1 (3)

**Table 5:** Prognostic factors by univariate analysis and multivariate analysis.

Prognostic factors	Univariate analysis	Multivariate analysis	
	*p*	Relative risk (95% CI)	*p*
Male	0.55	—	—
Age (<40)	0.15	—	—
PS (0)	0.012	—	0.54
Number of involved organs (<3)	0.013	0.31 (0.15–0.65)	0.0019
Head and neck	0.28	—	—
Intra-abdomen	0.25	—	—
Retroperitoneum	0.17	—	—
Lung	0.34	—	—
Liver	0.30	—	—
Bone	0.60	—	—
Lymph node	0.84	—	—
No bulky mass (<5 cm)	0.009	0.27 (0.12–0.60)	0.0013
Pretreatment	0.73	—	—
Normal, N/l	0.44	—	—
Normal Hb level (≧11.6 g/dl)	0.18	—	—
Normal LDH level (<222 mg/dl)	0.61	—	—
Normal ALP level (<322 IU/l)	0.12	—	—
Normal CRP level (<0.14 mg/dl)	0.020	0.43 (0.20–0.93)	0.032
